# Racial/Ethnic Patterns in Prostate Cancer Outcomes in an Active Surveillance Cohort

**DOI:** 10.1155/2011/234519

**Published:** 2011-06-26

**Authors:** Jennifer Cullen, Stephen A. Brassell, Yongmei Chen, Christopher Porter, James L'Esperance, Timothy Brand, David G. McLeod

**Affiliations:** ^1^Center for Prostate Disease Research, Department of Defense, Rockville, MD 20852, USA; ^2^Walter Reed Army Medical Center, Washington, DC, USA; ^3^Virginia Mason Medical Center, Seattle, WA, USA; ^4^Naval Medical Center San Diego, San Diego, CA, USA; ^5^Madigan Army Medical Center, Tacoma, WA, USA

## Abstract

*Introduction*. Concern regarding overtreatment of prostate cancer (CaP) is leading to increased attention on active surveillance (AS). This study examined CaP survivors on AS and compared secondary treatment patterns and overall survival by race/ethnicity. *Methods*. The study population consisted of CaP patients self-classified as black or white followed on AS in the Center for Prostate Disease Research (CPDR) multicenter national database between 1989 and 2008. Secondary treatment included radical prostatectomy (RP), external beam radiation therapy or brachytherapy (EBRT-Br), and hormone therapy (HT). Secondary treatment patterns and overall survival were compared by race/ethnicity. *Results*. Among 886 eligible patients, 21% were black. Despite racial differences in risk characteristics and secondary treatment patterns, overall survival was comparable across race. RP following AS was associated with the longest overall survival. *Conclusion*. Racial disparity in overall survival was not observed in this military health care beneficiary cohort with an equal access to health care.

## 1. Introduction

Racial/ethnic disparity in cancer outcomes has been extensively studied. With respect to prostate cancer (CaP), poorer patient outcomes among black men have been attributed to more advanced disease at the time of detection, less aggressive initial treatment, lower socioeconomic status (SES), inadequate quality and access to care, and/or more aggressive biology of the disease [1–14]. However, not all studies indicate that disparities exist. A recent meta-analysis concluded that there were no differences in CaP-specific or overall survival for white versus black men after accounting for methodological flaws of individual studies [15]. Similarly, when examining the accuracy of Partin tables for black men, Heath et al. found that race was not an independent prognostic factor for CaP progression despite higher grade and prostate-specific antigen (PSA) levels at baseline for black men [16]. Additional research has shown that once factors such as SES and treatment patterns are taken into account, observed racial disparities disappear [7, 12].

Growing concern regarding overtreatment of CaP is leading to increased interest in active surveillance (AS) as an option for patients with “low” or “very low” risk CaP. The National Comprehensive Cancer Network recommends AS for patients with “very low risk” CaP and a life expectancy of less than 20 years or men with a life expectancy of less than 10 years whose cancers are considered “low risk” [17]. The clinical dilemma becomes discerning if, and when, to intervene with secondary treatment. Factors that determine whether CaP is low, intermediate, or high risk include PSA at time of diagnosis, biopsy Gleason sum, and clinical stage at time of presentation [18]. Therefore, with the growing interest and clinical use of AS, the goal of this study was to assess whether or not this practice carries similar risk among racial/ethnic groups. 

Given the possibility that survival disparities may be a consequence of treatment modality, we examined secondary treatment patterns during the survivorship period within a cohort of patients initially followed on AS to determine whether there are differences across race/ethnicity in the following endpoints: (1) secondary treatment patterns, (2) overall survival. 

## 2. Methods

### 2.1. Study Population

The study population was comprised of men enrolled in the institutional review board (IRB)-approved Center for Prostate Disease Research (CPDR) multicenter national database. A description of this cohort and related data collection activities has been described previously [19, 20]. The study sample was restricted to patients diagnosed with CaP between January 1, 1989, and December 31, 2008, and for whom initial treatment was AS. For the purposes of this study, AS was defined as the absence of treatment with curative intent for a minimum of 9 months following CaP diagnosis. Therefore, the study sample was restricted to patients with at least 9-month followup after CaP diagnosis in order to define primary treatment as AS. Only white and black patients were analyzed because of inadequate sample sizes in other racial/ethnic categories. Secondary treatment was categorized in the following manner: those who continued AS until the end of the study period (no secondary treatment); radical prostatectomy (RP); external beam radiation therapy or brachytherapy (EBRT-Br); or hormone therapy (HT) after 9 months on AS.

### 2.2. Demographics and Clinical Characteristics

As part of routine data collection activities of the CPDR multicenter national database, the following demographic and clinical data were recorded for each subject: age at CaP diagnosis, self-reported race (i.e., white, black), PSA at diagnosis (categorized as <10, 10–19.99, and ≥20 ng/mL), clinical T stage (T1-T2a, T2b, T2c, and T3-4), biopsy Gleason sum (2–6, 7, 8–10), number of comorbidities (categorized as 0, 1, 2, 3+), secondary treatment type (categorized as none, RP, EBRT-Br, and HT), and dates of medical services. Risk strata were estimated using the criteria of D'Amico et al [18]. This approach combines diagnostic PSA, clinical T stage, and biopsy grade into a single composite index in order to classify men into low-, intermediate-, and high-risk disease. This classification schema has been described previously [18]. In brief, low-risk patients are defined as those with the following clinical characteristics: clinical stage T1c or T2a; PSA ≤ 10 ng/mL; Gleason score ≤6. Intermediate risk patients are classified as those with clinical stage T2b; or Gleason = 7; or PSA > 10 and ≤20 ng/mL. Finally, high risk patients are those with clinical stage T2c; or PSA > 20 ng/mL; or Gleason score 8–10. 

### 2.3. Study Endpoints

The primary study endpoint was overall survival. As part of data abstraction, vital status was reviewed annually as part of ongoing patient followup. Patient vital status was confirmed by searching the national death index using social security number, birth date, and name of the patient at the medical center where he was consented and enrolled into the database study. A secondary study endpoint included time to secondary treatment, which was calculated as the time from diagnosis with CaP to the time of initiation of RP, EBRT-Br, or HT. For patients who did not receive secondary treatment, followup time is censored at the end of the study period.

### 2.4. Statistical Analysis

Descriptive statistics included measures of central tendency (i.e., mean, median) as well as measures of dispersion (i.e., standard deviation (SD) range). Student *t* tests were used to compute means in continuous patient characteristics, included age, PSA at diagnosis, and followup time. Patient characteristics were computed for the overall sample, as well as stratified for race and secondary treatment type. Mantel Haenszel chi-square tests were used to compare distributions of categorical variables by race and secondary treatment type.

Kaplan Meier (KM) unadjusted estimation curves were plotted to examine the relationships between (1) race and secondary treatment and (2) race and overall survival. KM estimation was also used to examine potential statistical interaction between race and secondary treatment in predicting overall survival patterns by producing a single KM curve for each racial group. Overall survival was then stratified by secondary treatment type.

Multivariable Cox proportional hazards modeling was used to examine overall survival, controlling for key demographics and clinical characteristics. A stratified analysis was then conducted to examine possible effect modification between race and secondary treatment stratum (*N* = 4) with time to overall survival as the dependent outcome. Hazard odds ratio (HOR) effect estimates and corresponding 95% confidence intervals (CI) are reported. All statistical tests are 2 sided (summary  alpha = 0.05), and the decision rule was based on value < 0.05. All statistical analysis was performed using SAS 9.2 (SAS Institute Inc., Cary, NC). 

## 3. Results

Descriptive characteristics of the study sample are summarized in Tables 1 and 2, stratified for race/ethnicity and secondary treatment type, respectively. There were a total of 886 eligible patients. Twenty-one percent of the sample was black. Median age, time to secondary treatment, and followup time were 70.2, 19.6 months (1.6 years), and 5.2 years, respectively. Over two-thirds of patients had diagnostic PSA  values < 10 ng/mL. Almost three-quarters of subjects (74%) had at least one comorbid condition at time of CaP diagnosis. Three-quarters of patients had clinical stage T1-T2a disease (74.8%). Biopsy Gleason sum was 2–6 for 73% of subjects. More than half of the study sample (51%) was ≥70 years of age, yet almost half (45.3%) continued AS for primary treatment throughout the study period. By D'Amico et al. risk strata, almost half of the patients were considered low risk (49.0%), while more than a quarter of patients (25.7%) were intermediate and high risk (25.3%) at time of CaP detection. For those receiving secondary treatment, 14.1% had RP, 21.7% had EBRT-Br, and 19.0% had HT. 

Bivariate comparisons of sample characteristics across race demonstrate important differences (Table 1). Black men had a significantly younger mean age at CaP diagnosis (65.3 versus 70.4 years; *P* < 0.0001), a greater proportion of diagnostic PSA ≥ 10 (43.2% versus 28.3%; *P* < 0.0001), a greater proportion of high-risk disease (34.2% versus 22.8%; *P* = 0.0023), and a greater proportion of secondary treatment by RP or EBRT-Br combined (50.5% versus 31.7%; *P* < 0.0001). 

Table 2 shows bivariate comparisons of sample characteristics across secondary treatment type. Patients who received RP were younger with a median age of 61 years, compared to 72, 70, and 74 years for AS only, EBRT-BR, and HT, respectively (*P* < 0.0001). Patients who received AS or RP had lower median diagnostic PSA values than those receiving EBRT-BR or HT (*P* < 0.0001). Patients who had RP were also less likely to have multiple comorbidities compared to the other treatment groups (*P* = 0.017). The secondary treatment groups with the most adverse clinical features were those who went on to receive EBRT-Br and HT (*P* < 0.0001). Those who continued to receive AS throughout the study period had a significantly shorter median followup time (*P* < 0.0001). White patients were more likely to continue using AS than black patients, whereas the latter were more likely to receive RP or EBRT-Br secondary to AS. Those who had AS-HT had significantly longer intervals between CaP diagnosis and secondary treatment (median = 35 months or 2.9 years), while those on AS-RP had the shortest interval (median = 14.9 months or 1.2 years). Interestingly, none of the black patients who received HT secondary to AS were in the youngest age group (<60 years) as compared to only 4 (6%) white patients. However, the sample of black men in this treatment stratum was very small (*n* = 26).

**Figure 1:**
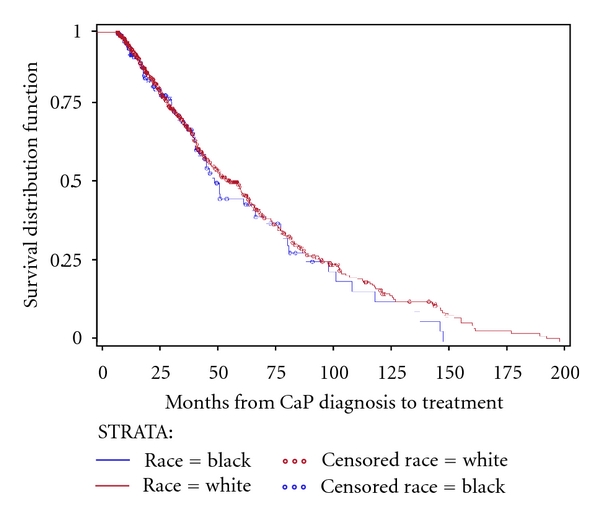
Kaplan Meier unadjusted estimation curve for time to secondary treatment stratified by race among subjects with prostate cancer (CaP) followed on active surveillance (AS) for primary treatment (*N* = 886).

**Figure 2:**
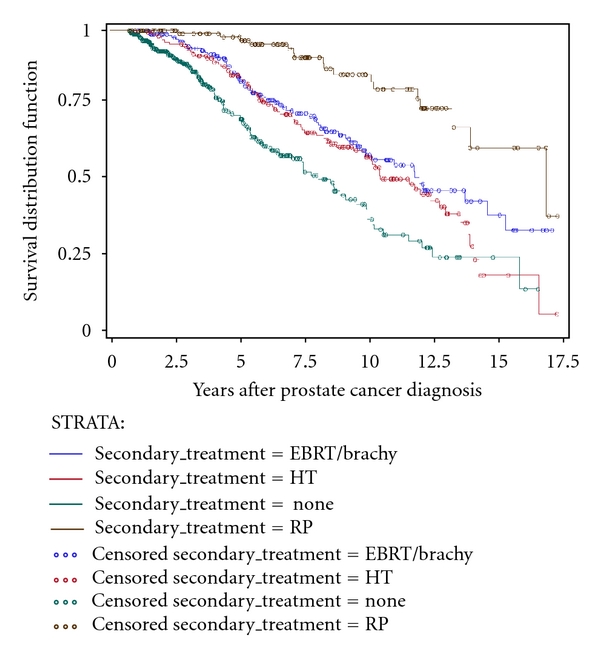
Kaplan Meier unadjusted estimation curve for overall survival stratified by secondary treatment type among subjects with prostate cancer (CaP) followed on active surveillance (AS) for primary treatment (*N* = 886).

**Figure 3:**
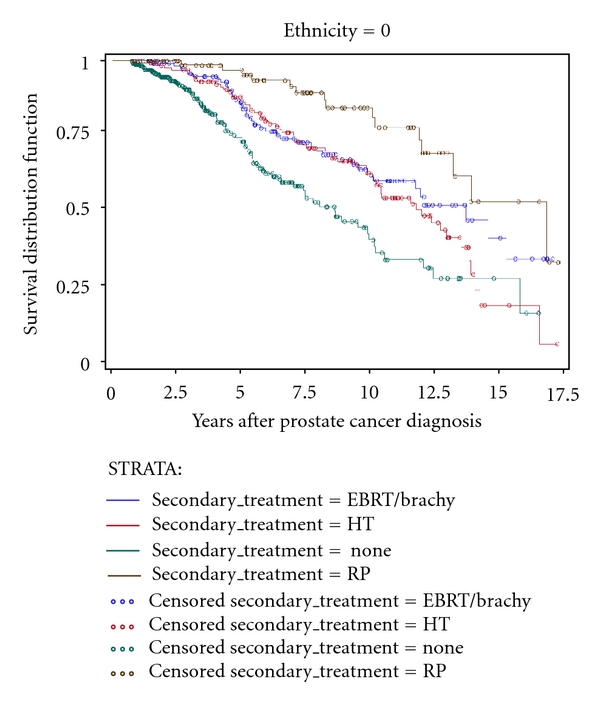
Kaplan Meier unadjusted estimation curve for overall survival among *white* men stratified by secondary treatment type among subjects with prostate cancer (CaP) followed on active surveillance (AS) for primary treatment (*n* = 696).

**Figure 4:**
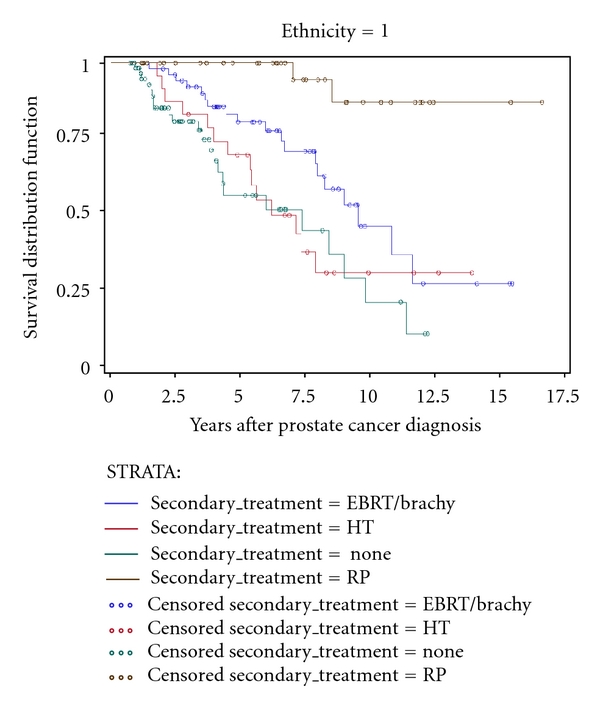
Kaplan Meier unadjusted estimation curve for overall survival among *black* men stratified by secondary treatment type among subjects with prostate cancer (CaP) followed on active surveillance (AS) for primary treatment (*n* = 190).

KM unadjusted time-to-event estimation curves are depicted in Figures 1–4. Time to secondary treatment was compared across race (Figure 1) revealing no statistically significant differences for black versus white patients; survival lines are parallel and roughly superimposed in the first 48 months after CaP diagnosis (log rank *P* = 0.42). Next, overall survival was examined as a function of secondary treatment type (Figure 2). This analysis was then repeated for black (Figure 3) and white (Figure 4) patients separately. Irrespective of race, a strong survival benefit was observed for patients receiving RP subsequent to AS versus all other secondary treatment groups (log rank *P* < 0.0001). In contrast, patients receiving AS only had the worst survival. 

Table 3 provides findings from multivariable Cox proportional hazards regression analysis predicting overall survival. This model shows that age at diagnosis (HOR_(≥70  versus  <60)_ = 1.9, CI = 1.03–3.36, *P* = 0.041), risk stratum (HOR_(High  versus  Low)_ = 2.6, CI = 1.93–3.58, *P* < 0.0001; HOR_(Intermediate  versus  Low)_ = 1.60, CI = 1.16–2.24, *P* = 0.0042), secondary treatment type (HOR_(RP  versus  None)_ = 0.022, CI = 0.011–0.043, *P* < 0.0001; HOR_(EBRT-Br  versus  None)_ = 0.052, CI = 0.031–0.087, *P* < 0.0001; HOR_(HTversus  None)_ = 0.107, CI = 0.069–0.167, *P* < 0.0001), and time from CaP diagnosis to secondary treatment (HOR_(per  month)_ = 0.97, CI = 0.965–0.976, *P* < 0.0001) were significantly associated with overall survival. 

Finally, multivariable Cox proportional hazards analysis predicting overall survival (Table 4) was conducted, stratified on secondary treatment type for a total of four models. These analyses show that, regardless of secondary treatment type, no racial disparity in overall survival was observed. Consistently across all 4 models, a significant predictor of overall survival was the D'Amico et al. risk classification. For three of four groups, this significant finding was restricted to comparison of risk at the extremes (i.e., high versus low). For the AS-only stratum, high D'Amico risk was associated with a 3.5 times increase odds of death from all causes (HOR_(High  versus  Low)_ = 3.52, CI = 2.18–5.69, *P* < 0.0001). Similarly, among the RP secondary treatment stratum, high D'Amico risk predicted more than a 5.5 increased odds of death (HOR_(High  versus  Low)_ = 5.64, CI = 1.48–21.4, *P* = 0.011); although the magnitude of this point estimate was large, it was also less precise due to a smaller sample size in this treatment group. For the EBRT-Br group, the risk comparison at the extremes was associated with over a fourfold increased odds of death (HOR_(High  versus  Low)_ = 4.20, CI = 1.98–8.90, *P* = 0.0002), while that for intermediate versus low risk demonstrated a borderline effect on survival, though it was not statistically significant: HOR_(Intermediate  versus  Low)_ = 2.16, CI = −5.3, *P* = 0.020 (Table 3). Finally, for the HT secondary treatment stratum, both comparisons of high versus low risk and intermediate versus low risk were significant in predicting overall survival: HOR_(High  versus  Low)_ = 2.6, CI = 1.4–4.8, *P* = 0.0022; HOR_(Intermediate  versus  Low)_ = 2.3, CI = 1.2–4.5, *P* = 0.018, respectively. 

Time from diagnosis with CaP to secondary treatment was also examined in the three relevant treatment groups: AS-RP, AS-EBRT-Br, and AS-HT. For both EBRT-Br and HT as secondary treatments, there was a statistically significant effect of this time interval on overall survival such that shorter time to treatment with curative intent from EBRT-Br and HT was associated with a slightly greater odds of death from all causes (HOR_(per  month)_ = 0.98, CI = 0.97–1.00, *P* = 0.042) and (HOR_(per  month)_ = 0.98, CI = 0.98–0.99, *P* = 0.0010), respectively. 

Multivariable Cox proportional hazards modeling was also performed stratified being by race with secondary treatment included as a model covariate in place of race, with comparable covariates entered into the secondary treatment-stratified models. In these 2 race-specific models, the lowest odds of death was observed for those who initiated RP secondary to AS for both black patients (HOR = 0.063, CI = 0.014–0.29, *P* = 0.0004) and white patients (HOR = 0.26, CI = 0.14–0.46, *P* < 0.0001); this effect was more pronounced in black patients (data not shown).

## 4. Discussion

Being black was not a predictor of poorer overall survival among participants of the CPDR multicenter national database undergoing AS as initial followup for CaP. This finding was evident despite clear racial differences in clinical characteristics at time of CaP detection. Specifically, black men were observed to have a greater proportion of intermediate- and high-risk disease, but this finding did not translate into longer-term adverse outcomes in terms of overall survival. 

Interestingly, for men who underwent secondary treatment, a striking benefit was observed among the group who received RP when controlling for key clinical characteristics. Men who remained on AS had the worst survival, despite controlling for baseline risk characteristics. This is especially striking given that these patients had the shortest median followup time of only 3.4 years. This may be explained, in part, by reduced intervention with additional treatments among patients for whom death seems imminent. This is supported by the finding that patients who remained on AS, only, were more likely to have 3 or more comorbid conditions at time of CaP diagnosis. 

Racial disparity in outcomes for prostate cancer survivors has been observed in several national data sources [7, 14, 21, 22]. In contrast, a recent meta-analysis concluded that there were no differences in overall or CaP-specific mortality for black versus white men with CaP [15]. Where racial differences have been noted, some researchers have proposed that variation in treatment patterns for CaP can be linked to a man's SES which in turn, may be partly to blame for observed racial disparities [4, 5, 9, 12].

Another possible explanation for racial disparity in CaP outcomes may be the geographical location or institution where health care services are received. Onega et al. found that higher overall mortality among black versus white Medicare beneficiaries was no longer significant when restricting analysis to location of services at the National Cancer Institute cancer centers. This finding lends support to the concept that place of services may, in part, account for observed racial differences [10]. 

Using the Detroit Surveillance, Epidemiology, and End Results data, Powell et al. found larger average tumor volumes in black versus white men after RP as well as a 4-fold ratio of distant disease among black versus white men. The authors conclude that these findings may indicate biological differences in disease progression [11].

In 2003, an Institute of Medicine report dedicated to the topic of unequal treatment in health care in the United States found that clear and striking differences exist in the receipt of services by race/ethnicity [23]. Other researchers have noted inequity in quality and type of care by race/ethnicity as a potentially contributing cause of disparities in CaP and overall survival [2, 6]. 

In an examination of CaP patients of African ancestry from New York, Guyana, and the Republic of Tobago and Trinidad, Mutetwa et al. found sharp survival rate disparity between Caribbean-born men diagnosed with CaP versus New York residents. However, immigrant Caribbean-born men had survival rates that approximated those of men from New York [8]. These findings argue for the importance of environmental factors in influencing outcomes for CaP survivors. This finding could include early detection of CaP, SES and receipt of treatment, location of health care services, and other factors not yet elucidated. When examining the interrelationships between race, SES, and treatment, Schwartz et al. found that much of the survival disadvantage for black men could be explained by a combination of low SES and receipt of nonsurgical treatment for disease [12].

In our study, we examined military health care beneficiaries participating in the CPDR multicenter national database. Patients in the CPDR database study constitute a screened cohort with regular PSAs and digital rectal examinations, in conjunction with annual physical examination beginning at age 40. Therefore, lack of racial/ethnic disparity in overall survival in this study sample may be, in part, attributable to accessibility to health care services. In the face of poorer baseline risk profiles among our black subjects, the observation of comparable survival outcomes may be explained by the shorter time to secondary treatment among black men, coupled with the preferential choice of RP secondary to AS among black. This explanation is consistent with our finding that the best overall survival was observed among men who received RP after AS.

### 4.1. Study Considerations

Despite important work that underscores the importance of SES in the relationship between race and survival, the CPDR does not systematically collect data on income or education. The closest correlation of SES in the CPDR cohort would be a patient's military rank, which was not available for this study. Albeit, patients included in this study are those eligible for military health care regardless of their education, income, or region of the country in which they receive services. While SES cannot be ruled as out as an explanatory factor in the absence of racial disparities in this cohort, we believe there is relative homogeneity with respect to SES in our cohort regardless of race.

A clear advantage to this study is the proportion of black men included. The CPDR database has an over-representation of black men—roughly 20%—compared to a 2010 national average of 13.5% [24]. As mentioned, we could not examine other racial/ethnic minorities such as Asian/Pacific Islanders and Hispanics as sample sizes because these groups are not large enough in the CPDR database to model the study endpoints of interest.

The key strengths of this study are the CPDR multicenter national database cohort itself, which contains a large proportion of black patients. Also, this cohort is coupled with long-term followup of its enrollees and strong adherence to receipt of care within the equal-access military health care system. These factors make the CPDR multicenter national database an excellent resource in which to examine racial patterns in CaP outcomes.

### 4.2. Future Directions

Further investigation is needed to explore why younger black men with higher-risk disease are opting for AS for initial treatment. Furthermore, we need a better understanding of what influences secondary treatment decisions. In spite of disparities in secondary treatment choices, study outcomes among patients receiving AS for primary treatment did not differ across race, despite racial differences in baseline clinical risk characteristics. 

Subsequent work in this expanding cohort of men will examine the specific patterns of health care delivery and use with regard to CaP. Studies of this nature will allow us, over time, to better understand how military health care beneficiaries are diagnosed and treated in our equal-access system after a CaP diagnosis.

## Figures and Tables

**Table 1:** Characteristics of subjects with prostate cancer (CaP) followed on active surveillance (AS) for primary treatment, stratified by race (*N* = 886).

Race characteristic	Total	White	Black	*P* value
	*N* = 886	*n* = 696	*n* = 190	
Age at diagnosis, years				<0.0001
Mean (±SD)^1^	69.3 (±8.4)	70.4 (±8.1)	65.3 (±8.4)	
Median (range)	70.2 (41.3–91.8)	71.7 (41.3–91.8)	65.6 (41.7–85.3)	
<60	109 (12.3)	67 (9.6)	42 (22.1)	
60–60.9	324 (36.6)	232 (33.3)	92 (48.4)	
≥70	453 (51.1)	397 (57.0)	56 (29.5)	
PSA at diagnosis, ng/mL, *N* (%)				<0.0001
<10	607 (68.5)	499 (71.7)	108 (56.8)	
10–19.99	153 (17.3)	115 (16.5)	38 (20.0)	
≥20	126 (14.2)	82 (11.8)	44 (23.2)	
Comorbidities, *N* (%)				0.1793
0	231 (26.1)	187 (26.9)	44 (23.2)	
1	264 (29.8)	205 (29.4)	59 (31.0)	
2	198 (22.3)	146 (21.0)	52 (27.4)	
≥3	193 (21.8)	158 (22.7)	35 (18.4)	
Clinical T stage, *N* (%)				0.1260
T1-T2a	660 (74.5)	520 (74.7)	140 (73.7)	
T2b	96 (10.8)	82 (11.8)	14 (7.4)	
T2c	68 (7.7)	49 (7.0)	19 (10.0)	
T3-4	62 (7.0)	45 (6.5)	17 (8.9)	
Biopsy grade, *N* (%)				0.1806
2–6	646 (72.9)	517 (74.3)	129 (67.9)	
7	168 (19.0)	127 (18.2)	41 (21.6)	
8–10	72 (8.1)	52 (7.5)	20 (10.5)	
D'Amico et al. risk strata, *N* (%)				0.0023
Low	434 (49.0)	359 (51.6)	75 (39.5)	
Intermediate	228 (25.7)	178 (25.6)	50 (26.3)	
High	224 (25.3)	159 (22.8)	65 (34.2)	
Secondary treatment type, *N* (%)				<0.0001
None (AS only)	401 (45.3)	333 (47.8)	68 (35.8)	
RP^2^	125 (14.1)	87 (12.5)	38 (20.0)	
EBRT-Br^3^	192 (21.7)	134 (19.2)	58 (30.5)	
HT^4^	168 (19.0)	142 (20.4)	26 (13.7)	
Time from Dx^5^to secondary treatment, months				0.0135
Mean (±SD)	30.6 (±26.6)	32.7 (±28.5)	24.5 (±18.6)	
Median (range)	19.6 (9.0–149.6)	20.3 (9.0–149.6)	16.0 (9.0–92.0)	
Followup, years				0.4641
Mean (±SD)	6.1 (±4.0)	6.1 (±4.0)	5.8 (±3.7)	
Median (range)	5.2 (0.8–17.2)	5.2 (0.8–17.2)	5.4 (0.8–16.8)	

^1^SD: standard deviation.

^2^RP: radical prostatectomy.

^3^EBRT-BR: external beam radiation therapy and Brachytherapy, combined.

^4^HT: hormone therapy.

^5^Dx: diagnosis of CaP.

**Table 2:** Characteristics of subjects with prostate cancer (CaP) followed on active surveillance (AS) for primary treatment, stratified by secondary treatment type (*N* = 886).

Secondary treatment type characteristic	None (AS^1^ only)	AS + RP^2^	AS + EBRT/Br^3^	AS + HT^4^	*P* value
	*n* = 401	*n* = 125	*n* = 192	*n* = 168	
Age at diagnosis, years					<0.0001
Mean (±SD)^5^	70.4 (±8.0)	60.7 (±7.9)	69.1 (±7.0)	73.3 (±7.0)	
Median	71.7	61.3	69.5	74.4	
Range	41.5–91.3	41.3–77.2	48.4–85.5	44.4–91.8	
PSA at diagnosis, ng/mL, *N* (%)					<0.0001
<10	322 (80.3)	98 (78.4)	101 (52.6)	86 (51.2)	
10–19.9	49 (12.2)	13 (10.4)	51 (26.6)	40 (23.8)	
≥20	30 (7.5)	14 (11.2)	40 (20.8)	42 (25.0)	
Race, *N* (%)					<0.0001
White	333 (83.0)	87 (69.6)	134 (69.8)	142 (84.5)	
Black	68 (17.0)	38 (30.4)	58 (30.2)	26 (15.5)	
Comorbidities, *N* (%)					0.0172
0	106 (26.4)	40 (32.0)	48 (25.0)	37 (22.0)	
1	115 (28.7)	40 (32.0)	56 (29.2)	53 (31.6)	
2	76 (19.0)	30 (24.0)	55 (28.6)	37 (22.0)	
3 or above	104 (25.9)	15 (12.0)	33 (17.2)	41 (24.4)	
Clinical T stage, *N* (%)					<0.0001
T1-T2a	330 (82.3)	92 (73.6)	129 (67.2)	109 (64.9)	
T2b	38 (9.5)	19 (15.2)	22 (11.5)	17 (10.1)	
T2c	21 (5.2)	10 (8.0)	15 (7.8)	22 (13.1)	
T3-4	12 (3.0)	4 (3.2)	26 (13.5)	20 (11.9)	
Biopsy grade, *N* (%)					<0.0001
2–6	318 (79.3)	99 (79.2)	119 (62.0)	110 (65.5)	
7	61 (15.2)	21 (16.8)	47(24.5)	39 (23.2)	
8–10	22 (5.5)	5 (4.0)	26 (13.5)	19 (11.3)	
D'Amico et al. risk strata					<0.0001
Low	246 (61.4)	68 (54.4)	61 (31.8)	59 (35.1)	
Intermediate	93 (23.2)	33 (26.4)	56 (29.2)	46 (27.4)	
High	62 (15.5)	24 (19.2)	75 (39.1)	63 (37.5)	
Time from Dx^6^ to secondary treatment, months					<0.0001
Mean (±SD^5^)	—	21.8 (±18.7)	25.7 (±21.3)	42.8 (±32.1)	
Median	—	14.0	16.7	34.8	
Range	—	9.0–121.2	9.0–115.0	9.2–149.6	
Followup, years					<0.0001
Mean (±SD^5^)	4.2 (±3.1)	7.6 (±4.3)	7.2 (±3.9)	7.9 (±3.8)	
Median	3.4	7.4	6.4	7.5	
Range	0.7–16.5	0.8–17.2	0.8–17.0	0.9–17.2	

^1^AS: active surveillance.

^2^RP: radical prostatectomy.

^3^EBRT-BR: external beam radiation therapy and Brachytherapy, combined.

^4^HT: hormone therapy.

^5^SD: standard deviation.

^6^Dx: diagnosis of CaP.

**Table 3:** Multivariable Cox proportional hazards regression predicting overall survival in a cohort of subjects with prostate cancer (CaP) followed on active surveillance (AS) for primary treatment (*N* = 886).

Characteristic	HOR^1^ (95% CI^2^)	*P* value
Age at diagnosis, years		0.1122
<60	Referent	—
60–60.9	1.837 (1.016–3.322)	0.0441
≥70	1.856 (1.026–3.357)	0.0408
Race		
White	Referent	—
Black	1.106 (0.805–1.519)	0.5362
Comorbidities		0.2714
0	Referent	—
1	1.235 (0.877–1.738)	0.2271
2	1.029 (0.700–1.512)	0.8847
3 or more	1.373 (0.952–1.978)	0.0895
D'Amico et al. risk strata		<0.0001
Low	Referent	—
Intermediate	1.612 (1.162–2.237)	0.0042
High	2.627 (1.927–3.580)	<0.0001
Secondary treatment type		<0.0001
None (AS only)	Referent	—
RP^3^	0.022 (0.011–0.043)	<0.0001
EBRT-Br^4^	0.052 (0.031–0.087)	<0.0001
HT^5^	0.107 (0.069–0.167)	<0.0001
Dx^6^ to secondary treatment, months	0.970 (0.965–0.976)	<0.0001

^1^HOR: hazard Odds Ratio.

^2^CI: confidence Interval.

^3^RP: radical prostatectomy.

^4^EBRT-BR: external beam radiation therapy and Brachytherapy, combined.

^5^HT: hormone therapy.

^6^Dx: diagnosis of CaP.

**Table 4:** Multivariable Cox proportional hazards model predicting overall survival in a cohort of subjects with prostate cancer (CaP) followed on active surveillance (AS) for primary treatment, stratified by secondary treatment type.

Secondary treatment type characteristic	None (AS only)		RP^3^		EBRT-Br^4^		HT^5^	
	HOR^1^ (95% CI^2^)	*P* value	HOR (95% CI)	*P* value	HOR (95% CI)	*P* value	HOR (95% CI)	*P* value
Age at diagnosis, years		0.1737		0.1532		0.3743		0.4236
<60	Referent	—	Referent	—	Referent	—	Referent	—
60–60.9	2.43 (0.84–6.97)	0.0983	1.65 (0.35–7.60)	0.5196	1.35 (0.45–4.02)	0.5836	1.84 (0.40–8.27)	0.4264
≥70	2.700 (0.95–7.64)	0.0616	4.41 (0.85–22.7)	0.0762	1.84 (0.62–5.42)	0.2663	1.32 (0.30–5.74)	0.7108
Race								
White	Referent	—	Referent	—	Referent	—	Referent	—
Black	1.24 (0.74–2.07)	0.4120	0.46 (0.09–2.32)	0.3522	1.14 (0.64–2.03)	0.6478	1.17 (0.60–2.29)	0.6282
Comorbidities		0.3218		0.4464		0.0061		0.5145
0	Referent	—	Referent	—	Referent	—	Referent	—
1	1.15 (0.66–1.99)	0.6059	2.42 (0.65–8.99)	0.1865	0.58 (0.27–1.23)	0.1582	1.06 (0.53–2.12)	0.8657
2	1.60 (0.85–3.02)	0.1425	0.97 (0.19–4.84)	0.9731	0.48 (0.21–1.06)	0.0724	1.22 (0.60–2.46)	0.5730
≥3	1.53 (0.86–2.71)	0.1431	2.05 (0.33–12.6)	0.4361	1.70 (0.82–3.53)	0.1518	0.71 (0.34–1.50)	0.3800
D'Amico et al. risk strata		<0.0001		0.0353		0.0007		0.0787
Low	Referent	—	Referent	—	Referent	—	Referent	—
Intermediate	1.47 (0.88–2.45)	0.1371	1.78 (0.44–7.12)	0.4124	2.16 (0.99–4.71)	0.0528	1.75 (0.87–3.51)	0.1112
High	3.52 (2.18–5.69)	<0.0001	5.64 (1.48–21.4)	0.0110	4.20 (1.98–8.90)	0.0002	2.03 (1.09–3.78)	0.0257
Dx^6^ to secondary treatment, months	—	—	1.00 (0.97–1.03)	0.8635	0.98 (0.97–1.00)	0.0424	0.98 (0.975–0.99)	0.0010

^1^HOR: hazard Odds Ratio.

^2^CI: confidence Interval.

^3^RP: radical prostatectomy.

^4^EBRT-BR: external beam radiation therapy and Brachytherapy, combined.

^5^HT: hormone therapy.

^6^Dx: diagnosis of CaP.
